# Correction: Praeruptorin B improves diet-induced hyperlipidemia and alleviates insulin resistance *via* regulating SREBP signaling pathway

**DOI:** 10.1039/c9ra90010a

**Published:** 2019-02-19

**Authors:** Zu-Guo Zheng, Chong Lu, Pyone Myat Thu, Xin Zhang, Hui-Jun Li, Ping Li, Xiaojun Xu

**Affiliations:** State Key Laboratory of Natural Medicines, China Pharmaceutical University 210009 Nanjing Jiangsu China xiaojunxu2000@163.com; Jiangsu Key Laboratory of Drug Discovery for Metabolic Diseases, China Pharmaceutical University 210009 Nanjing Jiangsu China

## Abstract

Correction for ‘Praeruptorin B improves diet-induced hyperlipidemia and alleviates insulin resistance *via* regulating SREBP signaling pathway’ by Zu-Guo Zheng *et al.*, *RSC Adv.*, 2018, **8**, 354–366.

The authors regret that there was an error in the corresponding authors indicated in the original article. There should only be one corresponding author, as indicated above.

The Royal Society of Chemistry apologises for these errors and any consequent inconvenience to authors and readers.

## Supplementary Material

